# Analysis and remedy of negativity problem in hybrid stochastic simulation algorithm and its application

**DOI:** 10.1186/s12859-019-2836-z

**Published:** 2019-06-20

**Authors:** Minghan Chen, Yang Cao

**Affiliations:** Department of Computer Science, Virginia Tech, Blacksburg, 24061 VA USA

**Keywords:** Hybrid stochastic algorithm, Negative population, Second slow reaction firing time

## Abstract

**Background:**

The hybrid stochastic simulation algorithm, proposed by Haseltine and Rawlings (HR), is a combination of differential equations for traditional deterministic models and Gillespie’s algorithm (SSA) for stochastic models. The HR hybrid method can significantly improve the efficiency of stochastic simulations for multiscale biochemical networks. Previous studies on the accuracy analysis for a linear chain reaction system showed that the HR hybrid method is accurate if the scale difference between fast and slow reactions is above a certain threshold, regardless of population scales. However, the population of some reactant species might be driven negative if they are involved in both deterministic and stochastic systems.

**Results:**

This work investigates the negativity problem of the HR hybrid method, analyzes and tests it with several models including a linear chain system, a nonlinear reaction system, and a realistic biological cell cycle system. As a benchmark, the second slow reaction firing time is used to measure the effect of negative populations on the accuracy of the HR hybrid method. Our analysis demonstrates that usually the error caused by negative populations is negligible compared with approximation errors of the HR hybrid method itself, and sometimes negativity phenomena may even improve the accuracy. But for systems where negative species are involved in nonlinear reactions or some species are highly sensitive to negative species, the system stability will be influenced and may lead to system failure when using the HR hybrid method. In those circumstances, three remedies are studied for the negativity problem.

**Conclusion:**

The results of different models and examples suggest that the Zero-Reaction rule is a good remedy for nonlinear and sensitive systems considering its efficiency and simplicity.

## Background

The stochastic simulation algorithm (SSA), also often called Gillespie’s algorithm [1, 2], is a major stochastic simulation method for simulating stochastic effects in biochemical networks. Although the SSA is quite reliable in numerous applications in computational biology, the algorithm is computationally intensive and inefficient for systems with fast reactions or large populations. Though many optimizations have been proposed to improve the efficiency of the algorithm [3–8], the essential idea of simulating each reacting event in a dynamical system makes it unpromising for large and complex biochemical systems compared to traditional deterministic methods.

To avoid the expensive computational cost of the SSA, researchers began studying approximation strategies. One well-known approximation is the *τ*-leap method [9], which approximates many reaction events in an interval of *τ* instead of simulating each reaction. As biological networks at single cell levels usually have large scale discrepancies in populations of species such as mRNAs and proteins, as well as rate constants among different reactions, research is increasingly focused on hybrid methods targeting multiscale systems that contain species populations or reaction rates with widely varying scales [10–13]. One branch of the hybrid method is the piecewise deterministic Markov process [10, 14, 15], which mixes the deterministic evolution with random jumps. Under the SSA branch, one hybrid method is to combine the *τ*-leap algorithm and the SSA for multiscale features among species populations [13]. Species and corresponding reactions are partitioned into two sets based on their populations, one simulated by the SSA and the other simulated by the *τ*-leap method. In a multiscale system, fast reactions can reach partial equilibrium or quasi-steady-state under certain conditions. Hybrid methods, like the slow-scale SSA method (ssSSA) [16, 17] and the stochastic quasi-steady-state SSA method (SQSSA) [12], were proposed based on this property. The ssSSA partitions the system into fast reaction and slow reaction sets, assuming partial equilibrium for the fast reactions, while simulating the slow reactions with the SSA. Similarly, the SQSSA first separates intermediate species and their corresponding reactions from the system, then assumes that the separated subsystem is at a steady state and simulates the rest of the system with the SSA. But both methods have limitations on parameter space to ensure the system validity [18, 19].

For general cases where fast reactions do not always reach a steady state or partial equilibrium, Haseltine and Rawlings [11] proposed a hybrid method (hereafter referred to as the HR hybrid method), which modeled part of the system by continuous dynamics (ordinary differential equations (ODEs) or Langevin equations), while keeping the rest discrete. The idea of the HR hybrid method was further explored, improved, and extended to several hybrid methods [20–23]. In Salis et al.’s work [20], they partitioned the system into fast and slow reaction groups, and modeled the fast group by Langevin equations and the slow group by the SSA. Later, Liu et al. [21] improved the efficiency of the HR hybrid method by a different partitioning strategy: reactions that have both low-density reactants and small reaction rates were put into the slow reaction subsystem and all the other reactions into the fast reaction subsystem. Wang et al. [24] optimized the implementation efficiency for the HR hybrid method and compared the efficiencies of the hybrid method coupled with three traditional ODE solvers RADAU5, DASSL, and DLSODAR. Lecca et al. [22] further divided the system into three sets: fast reactions, moderate reactions, and slow reactions, where the simulation of moderate reactions can be switched between stochastic and deterministic processes based on the reaction firing time during the system evolution. For spatial models or domains, hybrid methods were introduced under reaction-diffusion systems, where diffusion was approximated by differential equations to improve simulation efficiency [23, 25, 26].

Simulation tools, e.g. Hy3S [27] and MoBioS [22], and software like COPASI [28] were investigated based on the HR hybrid method and provided users with different simulation choices and implementation rules. As to the application on complex biochemical models, Wang et al. [29] used the HR hybrid method to model a budding yeast cell cycle. The method largely reduced the simulation time and the results matched well with experimental data on cell cycle properties and prototypes of most mutant cells. To mathematically analyze the accuracy of the HR hybrid method, Chen et al. [30] used the next reaction time of the slow reaction event as the accuracy benchmark and showed that the HR hybrid method is accurate in linear chain systems under certain conditions (either large populations of reactants in the fast subsystem or large scale differences of reaction rates between fast reactions and slow reactions). It also demonstrated that the HR hybrid method is valid for a much greater region in system parameter space than those for the ssSSA and the SQSSA methods.

However, in the HR hybrid method framework, populations of some reactant species may become negative if they are involved in both deterministic and stochastic systems. Take system (1) as an example. If reaction rate constants satisfy *f*_1_≫*k*_*c*_ and *b*_1_≫*k*_*c*_, the system can be divided into two groups: the fast reaction group and the slow reaction group, containing the reversible and irreversible reactions, respectively.

1 $$  \mathrm{S}_{1} \mathop{\rightleftharpoons}_{b_{1}}^{f_{1}} \mathrm{S}_{2} \overset{k_{c}}{\rightarrow} \mathrm{S}_{3}.  $$

Assume that this system has two S_1_ molecules at the beginning, and the system parameters are *f*_1_=1,*b*_1_=9,*k*_*c*_=0.01. Then, compared with the slow system, the fast system can be considered at equilibrium, which gives *x*_1_=1.8 and *x*_2_=0.2, where *x*_*i*_ denotes the mean population of species S_*i*_. Thus, when a slow reaction fires, *x*_2_ is reduced to − 0.8. Only after a certain period of time, it may become nonnegative again through the reaction S_1_→S_2_.

**Fig. 1:**
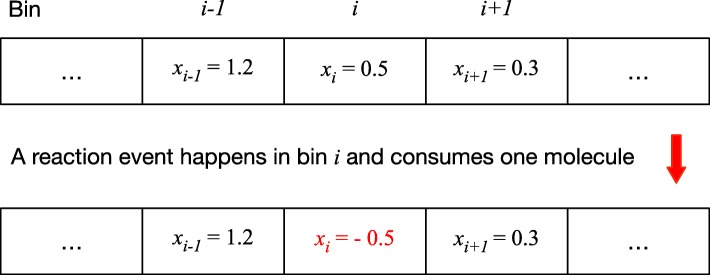
An example of a negativity phenomenon in a reaction diffusion system. *x*_*i*_ denotes the population of DivKp in the *i*th bin

Negative populations may also appear in stochastic simulations of reaction-diffusion systems, especially when low-density species are distributed in a well-meshed space. For example, in a one-dimensional spatial model of the *Caulobactor* cell cycle [31], the spatial domain is divided into 50 equally spaced bins. Since diffusion happens much faster than chemical reactions in the cell, diffusion events are modeled as continuous deterministic equations whereas chemical reactions are modeled by the SSA. In the initial stage of the cell cycle, protein DivKp has a low population (< 50). Thus the average population of DivKp inside each bin is < 1. Note that the mean population of a species is a real number if it is involved in the fast subsystem. As illustrated in Fig. 1, if there is a degradation reaction of DivKp firing in the *i*th bin, its population would become negative. Therefore, any consumption of those low population species in the spatial stochastic domain may lead to a negative population. The phenomenon of species’ populations becoming negative, as shown in the above two examples, is called the negativity problem for the HR hybrid method.

This paper is organized as follows. In the Methods section, we present the theoretical derivation of the second exit time of the chemical master equation (CME), the HR hybrid method, and three proposed remedies for the negativity problem. The Results and Discussion section analyzes the potential negativity effects on the accuracy of linear chain systems for a simple case (*n*=2) and a complex case (*n*=10). We test three remedies on three examples: a closed linear chain system, a nonlinear system, and a realistic biological system. Summary and conclusions are given last.

## Methods

### Second slow reaction firing time

Our prior work [30] analyzed the accuracy of the HR hybrid method by studying the next slow reaction firing time (NSRFT, also called the first exit time). Since the negative population problem mostly emerges after a slow reaction, it is not enough to just study the first exit time. So, we further extend that work and study the second slow reaction firing time (SSRFT, which can also be referred to as the second exit time). The SSRFT reflects the influence of a (possible) negative population on the firing of slow reactions. With this analysis, we hope to gain certain insight on the impact of the negativity problem on algorithm accuracy. In the HR hybrid method we assume reactants become negative only after a slow reaction happens, which is after the first exit time. Negative populations may also arise, with a much smaller probability, in the numerical integration of ODEs. That is not our focus here.

We use the same linear chain reaction network in Refs. [30, 32] as a study example, shown below.

2 $$ \mathrm{S}_{1} \mathop{\rightleftharpoons}^{f_{1}}_{b_{1}} \mathrm{S}_{2} \mathop{\rightleftharpoons}^{f_{2}}_{b_{2}} \cdots \mathop{\rightleftharpoons}^{f_{n-1}}_{b_{n-1}} \mathrm{S}_{n} \overset{k_{c}}{\rightarrow} \mathrm{S}_{n+1}.   $$

A particle can exit the reversible chain system through S_*n*_ with reaction rate *k*_*c*_. In most cases, *k*_*c*_ is comparably less than reaction rates *f*_*i*_ and *b*_*i*_ for 1≤*i*≤*n*−1. In many applications, the reversible chain reactions can be considered as a fast subsystem and the irreversible reaction (exit to S_*n*+1_) as a slow subsystem. With this partitioning strategy, if *x*_*n*_<1, then S_*n*_ will become negative whenever a slow reaction fires.

### SSRFT for the CME

While the first exit time (NSRFT) denotes the time when the next slow reaction fires in the linear chain system (2), the second slow reaction firing time (SSRFT) is the time period from the system start to the second time the slow reaction fires.

Recapping the derivation of NSRFT in Ref. [30], the SSRFT can be considered as the probability that two independent events (NSRFT) happen in a time interval [0, *t*]. In system (2), $\vec {x}(t)=(x_{1}(t)$, *x*_2_(*t*), …, *x*_*n*+1_(*t*))^*T*^ represents the system state at time *t*. If there is only one particle in the system, we denote the probability of *x*_*i*_=1 as

$$ p_{i}(t) = \mathrm{P}[x_{i}(t) = 1], \quad \text{ for } i = 1, \ldots, n+1. $$

and the probability vector for species S_1_,S_2_,…,S_*n*_ as

$$ \mathbb P(t) = [p_{1}(t), \ldots, p_{n}(t)]^{T} $$

In the chemical master equation system, we have

3 $$  \frac{d\mathbb P}{dt} = -A \mathbb P,  $$

where *A* is stoichiometric matrix,

$$ {{} \begin{aligned} A \,=\, \left[\!\! \begin{array} {c c c c c c} f_{1} & -b_{1} & 0 & 0 & \cdots & 0 \\ -f_{1} & b_{1} + f_{2} & -b_{2} & 0 & \cdots & 0 \\ 0 & -f_{2} & b_{2} + f_{3} & -b_{3} & \ddots & 0 \\ 0 & \ddots & \ddots & \ddots & \ddots & 0 \\ 0 & \cdots & 0 & -f_{n-2} & b_{n-2} + f_{n-1} & -b_{n-1} \\ 0 & \cdots & 0 & 0 & -f_{n-1} & b_{n-1} + k_{c} \\ \end{array} \!\right ]. \end{aligned}} $$

As there is only one particle all the time in the system, we have ${\sum \nolimits }_{i=1}^{n+1} p_{i}(t) = 1$. And *x*_*n*+1_(*t*)=1 if and only if the first exit time *T*_1_≤*t*. Thus

$$ \mathrm{P}[T_{1} \le t] = p_{n+1}(t) = 1 - \sum\limits_{i=1}^{n} p_{i}(t) = 1 - {\vec{e}}^{T} \mathbb P(t), $$

where $\vec {e} = [1, \ldots, 1]^{T}$. Given an initial condition $\vec {e}_{j}$ (a vector with the *j*th element equal to 1 and all other elements equal to 0), the NSRFT is (see Ref. [30])

$$ \begin{aligned} q_{j}(T_{1}) = \mathrm{P}[T_{1} > t]&= \vec{e}^{T} e^{-At} \vec{e}_{j} \\&= 1 - p_{n+1}(t) = 1\- \int_{0}^{t} k_{c} p_{n}(s) ds. \end{aligned} $$

In a general case where there are *m* particles ($\vec {x}_{0}=[m_{1}, m_{2}, \ldots, m_{n}]^{T}$, $m={\sum \nolimits }_{i=1}^{n} m_{i}$) in this linear system, as particles are independent of each other, the NSRFT is

$$ q(T_{1}) = \prod_{j=1}^{n} q_{j}^{m_{j}}(T_{1}). $$

Based on similar analysis to NSRFT, the second slow reaction firing time can be written as

4 $$ \begin{aligned} \mathrm{P}[T_{2} \le t] &= 1 - \prod_{j = 1}^{n} q_{j}^{m_{j}} \ - \sum\limits_{i=1, m_{i} \neq 0}^{n}\\&\quad C_{m_{i}}^{1} (1-q_{i}) q_{i}^{m_{i}-1}\prod_{j=1,j \neq i}^{n} q_{j}^{m_{j}}. \end{aligned}  $$

For a simple case where *n*=2 and the initial condition: *m*_1_=2 and *m*_2_=0, we have

5 $$ \mathrm{P}[T_{2} \le t] = \left (1-q_{1}(t) \right)^{2}.  $$

### SSRFT for the HR hybrid method

In the HR hybrid method, define the state vector in the fast subsystem as $\vec {x}(t) = [x_{1}(t), x_{2}(t), \ldots, x_{n}(t) ]^{T}$. The fast subsystem is modeled as a linear ODE system, denoted as

6 $$  \frac{d\vec{x}(t)}{dt} = -\tilde A \vec{x}(t).  $$

$\tilde A$ is a *n*×*n* matrix given by

$$ \tilde A = A - k_{c}\vec{e}_{n} \vec{e}_{n}^{T}, $$

where only the last elements of matrices $\tilde {A}$ and *A* are different, $\tilde {A}(n,n) = b_{n-1}$, *A*(*n*,*n*)=*b*_*n*−1_+*k*_*c*_.

Denote *T*_1_ as the NSRFT, we have

$$ \int_{0}^{T_{1}} k_{c}x_{n}(t) dt = \int^{T_{1}}_{0} \vec{e}_{n}^{T}e^{-\tilde At} \vec{x}_{0} dt = r, $$

where *r* is a unit exponential random number. In order to compare it with the CME result, we have to change the exponential random number to a unit uniform random number *u* by the relation *u*=1−*e*^−*r*^. The above equation can be written as

$$ \mathrm{P}[T_{1} \le t] = 1- e^{-\int_{0}^{T_{1}} k_{c} \vec{e}_{n}^{T} e^{-\tilde At} \vec{x}_{0} dt} = u. $$

And the density function of the NSRFT is

7 $$ p(T_{1}) = { k_{c} \vec{e}_{n}^{T} e^{-\tilde At} \vec{x}_{0} } e^{-\int_{0}^{T_{1}} k_{c} \vec{e}_{n}^{T} e^{-\tilde At} \vec{x}_{0} dt}.  $$

The SSRFT for the HR hybrid method can be considered as the next slow reaction firing time with a different initial condition $\vec {x}_{T_{1}}$ (the system state after the first exit time *T*_1_),

$$ \int_{T_{1}}^{T_{2}} k_{c} x_{n}(t) dt = \int_{T_{1}}^{T_{2}} \vec{e}_{n}^{T}e^{-\tilde A(t-T_{1})} \vec{x}_{T_{1}} dt = r, $$

where $\vec {x}_{T_{1}} = e^{-\tilde A T_{1}} \vec {x}_{0} - \vec {e}_{n}$. Thus

8 $$ \begin{aligned} p(T_{2}|T_{1}) &= {\left(k_{c} \vec{e}_{n}^{T} e^{-\tilde A(T_{2}-T_{1})} \vec{x}_{T_{1}} - k_{c} \vec{e}_{n}^{T} \vec{x}_{T_{1}} \right) }\\&\quad e^{-\int_{T_{1}}^{T_{2}} k_{c} \vec{e}_{n}^{T} e^{-\tilde A(t-T_{1})} \vec{x}_{T_{1}} d t}. \end{aligned}  $$

Therefore, the SSRFT is

9 $$ p(T_{2}) = \int_{0}^{\infty} p(T_{1})p(T_{2}|T_{1}) d T_{1}.  $$

Below we present three strategies to handle the negativity problem and compare the corresponding impact on the SSRFT.

### SSRFT for remedy I: zero-population

For the negativity problem in the HR hybrid method, one simple treatment is to immediately change any negative value to zero. So we name this strategy the **Zero-Population** remedy: after a slow reaction happens in the stochastic subsystem, detect whether any corresponding reactants become negative, if so set them as zero and continue the simulation, otherwise continue without modification.

To check the effect of the Zero-Population remedy, we study the second slow reaction firing time of the system under this rule. Since the rule takes effect after the first slow reaction, only the conditional density function *p*(*T*_2_|*T*_1_) is different from the HR hybrid method when *x*_*n*_<0. In this scenario, the initial condition of the second slow reaction firing time in linear chain system (2) is

10 $$~ \vec{x}_{T_{1}} = e^{-\tilde A T_{1}} \vec{x}_{0} - \vec{e}_{n}, \quad \vec{x}_{T1}(n) = \max(0,\ \vec{x}_{T1}(n)).  $$

The conditional density function of the second exit time in the Zero-Population remedy is similar to Eq. (8), just replace the initial condition with $\vec {x}_{T1}(n)$ in Eq. (10).

### SSRFT for remedy II: zero-reaction

While the Zero-Population remedy avoids the negative effect, it changes the conservation law in the system. For example, the total amount of all species in the closed system (2) should always be *m*, but simply changing the negative population (−*m*_*δ*_) to zero causes the total population to increase to *m*+*m*_*δ*_. In order to follow the law of conservation, which is important in many practical applications, one idea is to scale down a reaction when the slow reaction happens with a reactant population less than one. Take the reaction X → Y as an example. Suppose the population of reactant X is less than one (0<*m*_*X*_<1), then instead of consuming one molecule of X, scale the reaction by a ratio of *m*_*X*_, which produces *m*_*X*_ molecule of Y. However, this method breaks the natural discrete feature of slow reaction firing and can cause significant errors for stochastic models.

Alternatively, one can simply set all related reaction propensities as zero when a negative population appears. So we have the second remedy named the **Zero-Reaction** rule: set all reaction propensities involving negative species as zero in corresponding subsystems until the negative species become nonnegative.

After the first slow reaction, the system status is $\vec {x}_{T_{1}} = e^{-\tilde A T_{1}} \vec {x}_{0} - \vec {e}_{n}$. For *x*_*n*_<0, reaction rates for all reactions that S_*n*_ participated in the fast subsystem are treated as zero, and the corresponding coefficient matrix for the ODEs is denoted as $\hat A$. The propensity of the slow reaction, which S_*n*_ participated in, is also set to zero. Since system (2) has only one slow reaction, no reaction in the slow subsystem will fire for negative *x*_*n*_. The fast subsystem keeps running until *x*_*n*_=0. We denote *τ* as the time period of *x*_*n*_ evolving from negative to zero in the ODE system:

11 $$ \vec{e}_{n}^{T} e^{- \hat A \tau} \vec{x}_{T_{1}} = 0,  $$

where the above equation has a unique solution *τ*. So after time *T*_1_+*τ*, the system is back to normal, with the system state $\vec {x}_{\tau } = e^{-\hat A \tau }\vec {x}_{T_{1}}$.

Thus, the conditional density function of the SSNFT under the Zero-Reaction remedy is

12 $$ {\begin{aligned} p(T_{2}|T_{1}) &= { \left(k_{c} \vec{e}_{n}^{T} e^{-\tilde A(T_{2}-T_{1}-\tau)} \vec{x}_{\tau} - k_{c} \vec{e}_{n}^{T} \vec{x}_{\tau} \right)}\\&\quad e^{-\int_{T_{1}+\tau}^{T_{2}} k_{c} \vec{e}_{n}^{T} e^{-\tilde A(t-T_{1}-\tau)} \vec{x}_{\tau} d t}. \end{aligned}}  $$

### SSRFT for remedy III: zero-time

Another remedy for the negativity problem is called the **Zero-Time** rule: whenever a species’ population become negative, pause the system and run a separate virtual ODE system G*f*′ that only contains reactions related to the negative species. When the species in the virtual system recovers to a nonnegative state, restart the original hybrid system with the updated system state. Because the system recovers under existing reaction systems, the conservation law is obeyed.

In system (2), the system state is still $\vec {x}_{T_{1}} = e^{-\tilde A T_{1}} \vec {x}_{0} - \vec {e}_{n}$ after the first slow reaction. For *x*_*n*_<0, build a separate ODE system that only contains the last reversible reaction,

13 $$ \mathrm{G}'_{f}: \mathrm{S}_{n-1} \mathop{\rightleftharpoons}^{f_{n-1}}_{b_{n-1}} \mathrm{S}_{n}.  $$

Run the G*f*′ system until *x*_*n*_=0, which costs time *ρ* from the below equation.

14 $$ \vec{e}_{2}^{T} e^{- B \rho} \left [ \begin{array}{c} x_{n-1} \\ x_{n} \end{array} \right ] = 0, \quad B = \left [ \begin{array} {c c} f_{n-1} & -b_{n-1} \\ -f_{n-1} & b_{n-1} \end{array} \right ]  $$

Since the recovery time *ρ* is not counted in system evolution, we have the conditional density function of the second exit time under the Zero-Time remedy as

15 $$ {{} \begin{aligned} &p(T_{2}|T_{1}) =\\& { (k_{c} \vec{e}_{n}^{T} e^{-\tilde A(T_{2}-T_{1})} \vec{x}_{\rho} \,-\, k_{c} \vec{e}_{n}^{T} \vec{x}_{\rho}) } e^{-\int_{T_{1}}^{T_{2}} k_{c} \vec{e}_{n}^{T} e^{-\tilde A(t-T_{1})} \vec{x}_{\rho} d t}, \end{aligned}}  $$

where $\vec {x}_{\rho }$ is the system state after running the G*f*′ system for *ρ*.

## Results and discussion

### Theoretical analysis of SSRFT

#### A simple case (*n*=2)

This subsection examines the SSRFT of the linear system (2) with *n*=2, as shown in (1).

We first check conditions that may cause the negativity problem, such as parameters and initial conditions. In system (1), after the first slow reaction, we have

$$\vec{x}_{T_{1}} = e^{-\tilde A T_{1}} \vec{x}_{0} - \vec{e}_{2}, \quad \tilde A = \left [ \begin{array}{c c} f_{1} & -b_{1} \\ -f_{1} & b_{1} \end{array} \right ], $$

thus the population of S_2_ at time *T*_1_ is

$$\vec{x}_{T_{1}}(2) \,=\, \frac{(m_{2}b_{1}-m_{1}f_{1}) e^{- (b_{1} + f_{1})T_{1}} \ +(m_{1}+m_{2})f_{1}}{b_{1} + f_{1}}-1. $$

The first slow reaction firing may happen from time 0 to *∞*. To ensure that the population of S_2_ is nonnegative for all possible *T*_1_, we should have $\vec {x}_{T_{1}}(2) \ge 0$ for all *T*_1_. We solve this inequality and get

16 $$ m_{1} + m_{2} \ge \frac{f_{1}+b_{1}}{f_{1}} \quad \text{and} \quad m_{2} \ge 1.  $$

For the parameter set *f*_1_=*b*_1_=1, the population of S_2_ may become negative when the initial condition satisfies *m*_2_=0 (Assume *m*_1_+*m*_2_≥2, so a second slow reaction is possible). For the parameter set *f*_1_=1,*b*_1_=10, the population of S_2_ may become negative when the initial condition satisfies *m*_2_=0 or *m*_1_+*m*_2_<11.

**Fig. 2:**
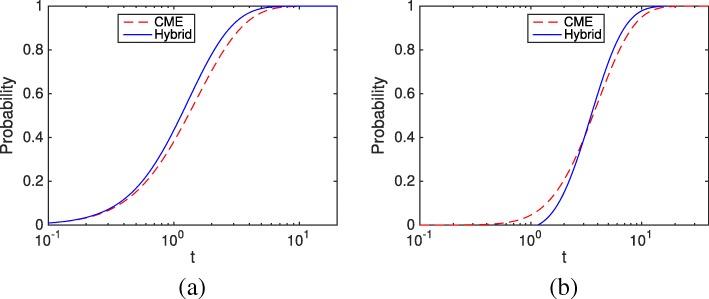
Cumulative probability distributions of NSRFT and SSRFT in the linear system (1) of the CME and the HR hybrid method. Parameters used in this example are: *f*_1_=*b*_1_=*k*_*c*_=1. The initial condition is *m*_1_=2,*m*_2_=0. **a** Cummulative probablitity distributions of NSRFT **b** Cummulative probablitity distributions of SSRFT

Figure 2 presents the cumulative distribution functions (CDFs) of NSRFT and SSRFT from both the CME and the HR hybrid method, respectively. The model parameters and initial conditions are chosen so that the negativity problem may arise. For the NSRFT, when *t* is small, the two methods are close enough. When *t* ∈[ 1,10], the HR hybrid method has the first slow reaction firing earlier than the CME. In this time interval, the mismatch between the two methods comes from the error of the hybrid method, rather than the negativity problem. For the SSRFT, the CDFs of two methods have an intersection at around *t*^∗^=2, where before this point the HR hybrid method tends to fire the second slow reaction later than the CME. This difference shows the impact of the negativity problem. After the first slow reaction, *x*_2_ becomes negative, and the system needs extra time to recover, which causes a delay for the SSRFT in the HR hybrid method.

For comparison, we calculate the relative error

17 $$ e_{r}=\frac{|T_{c}-T_{h}|}{T_{c}},  $$

where *T*_*c*_ and *T*_*h*_ are the second slow reaction firing times of the CME and the HR hybrid method, respectively. Here we sample the mean SSRFT as *T*_*c*_ and *T*_*h*_, i.e. *P*(*T*_2_<*T*_*c*_)=0.5, *P*(*T*_2_<*T*_*h*_)=0.5.

**Fig. 3:**
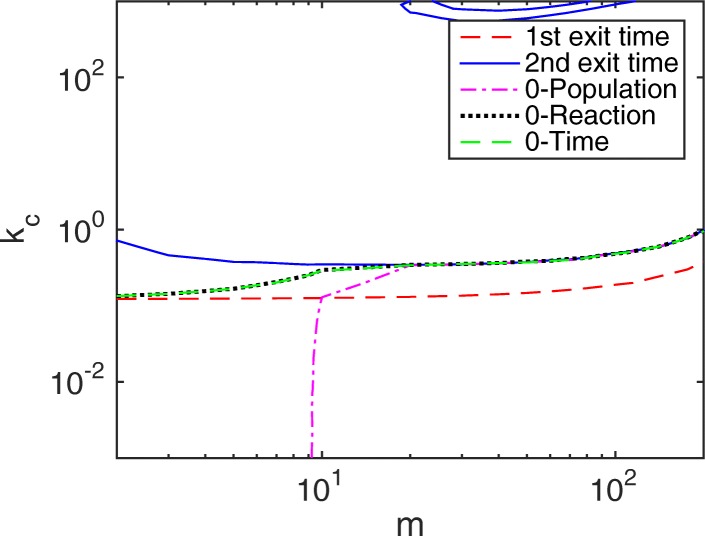
Contour plot of relative error *e*_*r*_ in the linear system (1) with parameters *f*_1_=1,*b*_1_=10. The initial condition is set to *m*_1_=*m*,*m*_2_=0. Regions below each line have a relative error less than 1%. For the Zero-Population rule, the bottom right region is the acceptable parameter space

When we increase *b*_1_ from 1 to 10 and set the initial condition as *m*_1_=*m*,*m*_2_=0, then S_2_ has a low population and a great chance to become negative when the slow reaction fires. In Fig. 3, the acceptable region for *e*_*r*_<0.01 of the SSRFT is surprisingly larger than that of the NSRFT. But it can be well explained. First, it is known that the NSRFT of the HR hybrid method is faster than that of the CME from the previous work [30] and the example in Fig. 2a. If there is no negativity problem, then the SSRFT of the hybrid method should be even faster than that of the CME as it accumulates error from two slow reactions. However, when there is a negative species, e.g. S_2_ in this case, the system slows down to recover from its negative state. The negativity recovery time, to some extent, reduces the numerical error of the hybrid method in the linear chain system. This runs like a way of coordination between two subsystems: when a species becomes negative caused by a slow reaction, i.e. the slow subsystem runs faster than expected, then the slow subsystem has to wait longer for the next slow reaction to fire again.

For the Zero-Reaction and Zero-Time remedies, though they have a smaller acceptable parameter space compared with the HR hybrid method, they still have a larger parameter region than the NSRFT. Note that in the linear chain system, the Zero-Time rule is similar to the Zero-Reaction rule as G*f*′ is the fast subsystem and time *τ*,*ρ* are comparably small, but they are different for other cases, such as when G*f*′ is different from the fast subsystem or when there are more slow reactions in the slow subsystems. For the Zero-Population remedy, the acceptable parameter space is only half of the other methods. All three remedies converge to the contour line of the HR hybrid method when *m*≈11, satisfying one of the nonnegative requirements (16). On the other hand, when the total population is large (or $m\ge \frac {f_{1}+b_{1}}{f_{1}}$), a negative population appears in a shorter time window, whereas Ref. [30] has demonstrated that the error of the HR hybrid method becomes smaller with larger populations. This leads us to a conjecture regarding the HR hybrid method for the linear chain reaction system: the error caused by negative populations is negligible compared to the original error of the hybrid method. In other words, the negativity effect is substantial only when the method is already problematic in accuracy. Sometimes it acts as a positive sign to reduce the numerical error of the HR hybrid method.

**Fig. 4:**
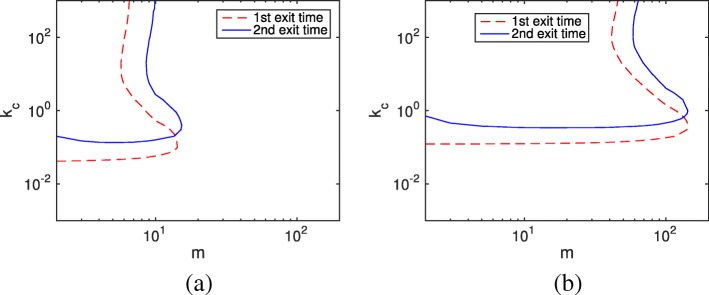
Contour plot of the average relative error *e*_*r*_=0.01 in the linear system (1) with different *k*_*c*_ and *m* values. The remaining parameter is *f*_1_=1. Acceptable parameter pairs for the HR hybrid method can be chosen from the bottom and right regions. **a** Contour plot of the average relative error *e*_*r*_=0.01 with parameter *b*_9_=1**b** Contour plot of the average relative error *e*_*r*_=0.01 with parameter *b*_9_=10

For general cases that include both negative and nonnegative situations, we calculate the mean relative error based on all possible initial conditions $\vec {x}_{0}$, which follows the steady state distribution of the fast subsystem subject to *f*_1_ and *b*_1_. Figure 4 illustrates the acceptable system parameter region (*e*_*r*_<0.01) for *b*_1_=1 and *b*_1_=10. In both cases, the SSRFT keeps the same pattern but with improved accuracy horizontally resulting from the negativity phenomenon and decreased accuracy vertically due to the accumulative method error. Since nonnegative situations occur much more frequently than negative situations (where the initial condition must be either *m*_2_=0 or *m*<11), the three proposed remedies do not make a difference in the acceptable region of the HR hybrid method.

#### A larger system (*n*=10)

If we increase the length of the linear chain system to *n* = 10, it is hard to calculate the derived formulas (4, 9) for the second exit time. Instead, we run simulations of each method and collect samples for the NSRFT and the SSRFT. For each pair of (*m*, *k*_*c*_), the reported NSRFT and SSRFT are the mean values from one million simulation results. Consider that all the contour plots in this subsection are processed to make the outline smooth.

**Fig. 5:**
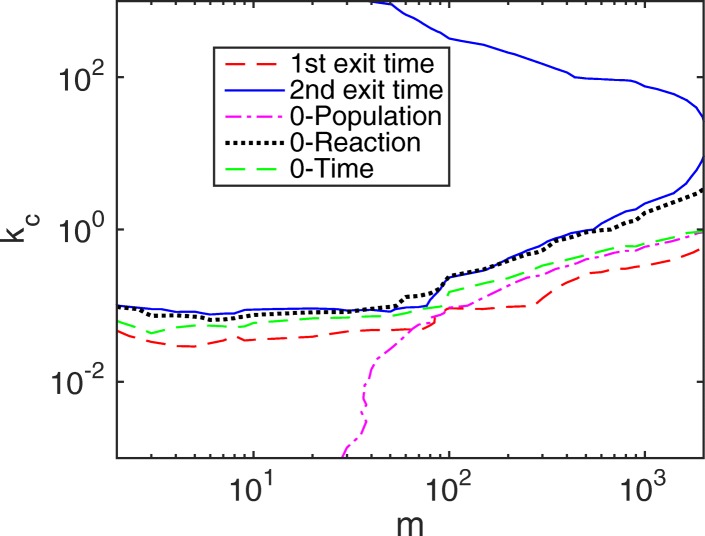
Contour plot of relative error of *e*_*r*_ in the linear chain system (2) with parameters *f*_*i*_=*b*_*i*_=*k*_*c*_=1,*b*_9_=10. The chain length is *n*=10 and the initial condition is set to *m*_1_=*m*,*m*_*i*_=0 (*i*=2,3,…,10). Regions below each line have a relative error less than 1%. Note that the HR hybrid method has an extra top right region of acceptable parameter pairs

First look at one negative case where the initial condition is *m*_1_ = *m*, *m*_*i*_ = 0 (*i* = 2,3,…,10). In Fig. 5, the shape of the acceptable region (*e*_*r*_ < 0.01) is similar to the case when *n*=2. For the Zero-Reaction rule, it has the same accuracy as the original HR hybrid method, except for the top right region; while the Zero-Population rule is only accurate for large *m* and small *k*_*c*_. So the observation that negativity does not influence the accuracy still holds for large linear chain systems, if the Zero-Reaction rule is applied. We do not consider the Zero-Time rule as it is much less efficient than the other two remedies in this larger system.

**Fig. 6:**
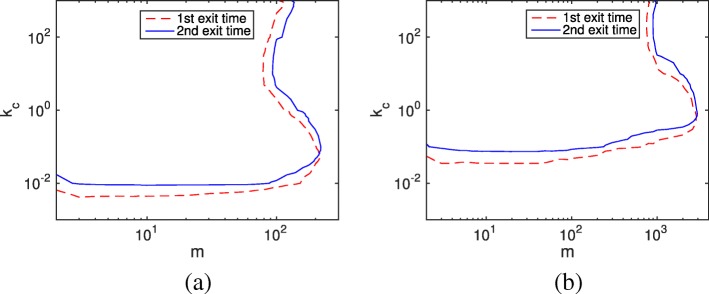
Contour plot of the average relative error *e*_*r*_=0.01 in the linear chain system (2) with different *k*_*c*_ and *m* values. The chain length is *n*=10. Acceptable parameter pairs for the HR hybrid method can be chosen from the bottom and right parts. **a** Contour plot of the average relative error *e*_*r*_=0.01 with parameter *b*_9_=1**b** Contour plot of the average relative error *e*_*r*_=0.01 with parameter *b*_9_=10

For general cases, we randomly sample initial conditions for each (*m*, *k*_*c*_) pair. And the probability distribution of $\vec {x}_{0}$ satisfies the steady state of the reversible reactions controlled by *f*_*i*_ and *b*_*i*_. Figure 6 exhibits the same pattern as Fig. 4 for both cases *b*_9_=1 and *b*_9_=10. The acceptable parameter space for the second exit time is smaller when *n*=10, which is similar to the first exit time discussed in Ref. [30]. Thus, for large linear chain systems, the negativity problem is insignificant for the hybrid method.

### Numerical experiments

The previous subsection studied the accuracy based on the first and the second slow reaction times. In this section, we apply the HR hybrid method and three remedies to different systems and compare statistics.

#### A closed linear chain system

The first example shown below is a similar linear chain system with an extra reaction $\mathrm {S}_{3} \overset {k_{s}}{\rightarrow } \mathrm {S}_{1}$ that forms a closed system. We divide the system into two parts, the ODE system contains the reversible reaction, while the SSA system takes the remaining reactions involving S_3_.

18 $$  \mathrm{S}_{1} \mathop{\rightleftharpoons}^{f_{1}}_{b_{1}} \mathrm{S}_{2} \overset{k_{c}}{\rightarrow} \mathrm{S}_{3} \overset{k_{s}}{\rightarrow} \mathrm{S}_{1}.  $$

**Fig. 7:**
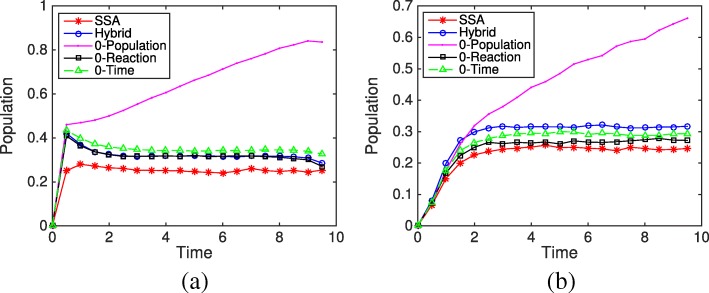
Evolution of species S_2_ and S_3_ in the closed linear system (18) from the SSA, the HR hybrid method, and three remedies (Zero-Population, Zero-Reaction, and Zero-Time) based on 10,000 simulations. The parameters are *f*_1_=*b*_1_=*k*_*c*_=*k*_*s*_=1. The initial condition is *m*_1_=1 and the remaining species populations are zero. **a** Population evolution of species S _2_**b** Population evolution of species S _3_

**Fig. 8:**
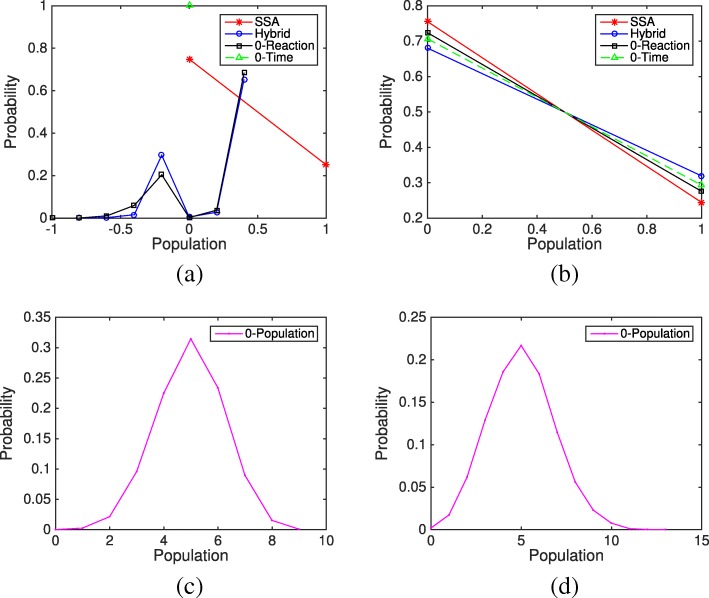
Final distributions of species S_2_ and S_3_ in the closed linear system (18) from the SSA, the HR hybrid method, and three remedies (Zero-Population, Zero-Reaction, and Zero-Time) based on 10,000 simulations. The parameters are *f*_1_=*b*_1_=*k*_*c*_=*k*_*s*_=1. The initial condition is *m*_1_=1 and the remaining species populations are zero. **a** Final population distributions of species S _2_**b** Final population distributions of species S _3_**c** Final population distribution of species S _2_ under the Zero-Population remedy **d** Final population distribution of species S _3_ under the Zero-Population remedy

We choose model parameters and initial conditions so that negative populations occur frequently in the system. Figure 7 shows the average evolution of species S_2_ and S_3_ from different simulation methods and rules over 10,000 simulations. It is obvious that under the Zero-Population rule, the populations of S_2_ and S_3_ keep increasing with time while the other methods reach a steady state. The evolution from the Zero-Reaction rule is slightly closer to the results of SSA than the other methods. We then look at the final distributions of species S_2_ and S_3_ based on 10,000 simulations, shown in Fig. 8. In system (18), *x*_2_ is negative for 30% of the time if using the original HR hybrid method. With the Zero-Time rule, *x*_2_ is always nonnegative, while the Zero-Reaction rule does not change the distribution of S_2_ much. For the final distribution of S_2_, though the differences between methods are fairly close (< 10*%*), the Zero-Reaction rule also works better than others. The results from the Zero-Population rule are much different from the SSA results, and the final distribution will shift further to the right if we run the system longer.

The above example demonstrates that the system can finally recover from negative populations without using any remedies. To test an extreme case, suppose there is another species Y highly sensitive to S_2_, shown below

19 $$ \mathrm{S}_{1} \mathop{\rightleftharpoons}^{f_{1}}_{b_{1}} \mathrm{S}_{2} \overset{k_{c}}{\rightarrow} \mathrm{S}_{3}, \quad \emptyset \overset{k_{1} [\mathrm{S}_{2}]}{\rightarrow} \mathrm{Y} \overset{k_{2}}{\rightarrow} \emptyset   $$

The first part is a simple linear chain system with *n*=2. A particle can exit the reversible chain system through S_2_ with reaction rate *k*_*c*_. The other part is a birth and death process of species Y. S_2_ acts as the enzyme activating the synthesis of Y. Following the same partition strategy used above, we isolate the irreversible reaction in a slow subsystem as both the rate constant *k*_*c*_ and quantity of S_2_ are small. All the remaining reactions are put into a fast subsystem.

**Fig. 9:**
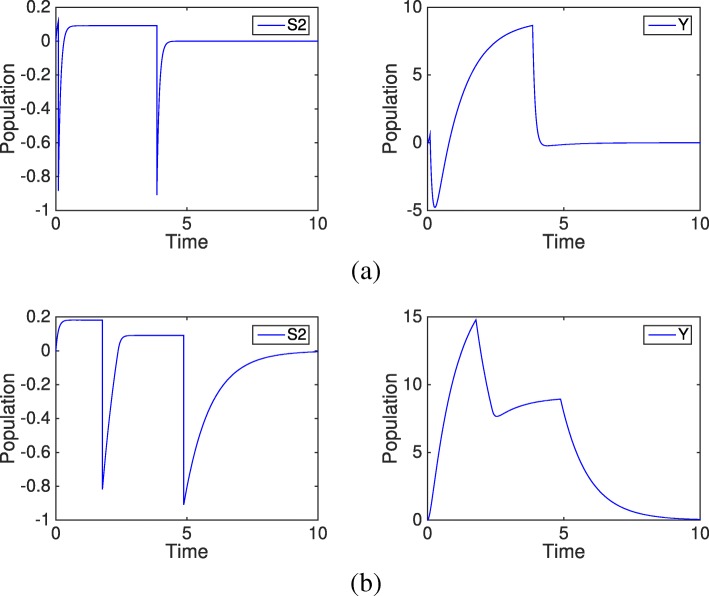
Population trajectories of S_2_ and Y in system (19) from one simulation of the HR hybrid method and the Zero-Reaction remedy. The parameters are *f*_1_=1,*b*_1_=10,*k*_*c*_=1,*k*_1_=100,*k*_2_=1. The initial condition is *m*_1_=2 and the remaining species populations are zero. **a** Population trajectories of species S _2_ and Y from one HR hybrid method simulation **b** Population trajectories of species S _2_ and Y from one Zero-Reaction remedy simulation

By setting *f*_1_/*b*_1_=0.1, S_2_ maintains a low population and has a high chance to become negative when the slow reaction fires. Once *x*_2_<0, the slow reaction propensity is negative, which ensures that the slow reaction will not fire until *x*_2_ goes back to a positive value in the fast subsystem. So the negative population of S_2_ has no effect on S_3_ in this case. But for Y, it can provoke large fluctuations especially when *k*_1_[S_2_]≫*k*_2_. Figure 9 shows one simulation result of S_2_ and Y. When S_2_ first drops to − 0.7, Y is also driven negative and then becomes positive after one time unit, while under the Zero-Reaction rule, Y is always nonnegative. During the recovery period of Y, if there is another species Z dependent on Y and a species A dependent on Z, then Y may incur a cascade of negativity which significantly increases the simulation error. For situations where negative reactants heavily affect other species, a remedy is definitely needed to prevent a simulation failure.

#### A closed nonlinear system

From the previous studies, we found that the HR hybrid method works fine for linear systems even with a high frequency of negative populations. Here we want to examine the effect of negative values on nonlinear systems, e.g. bimolecular reactions. Slightly modifying reactions involving S_2_ into bimolecular reactions in system (18), we generate a new nonlinear system shown below.

20 $$ \mathrm{S}_{1} \mathop{\rightleftharpoons}^{f_{1}}_{b_{1}} 2 \mathrm{S}_{2} \overset{k_{c}}{\rightarrow} \mathrm{S}_{3} \overset{k_{s}}{\rightarrow} \mathrm{S}_{1}.   $$

Similarly, we partition the system into groups: the fast group containing the reversible reactions and the slow group containing the remaining reactions. For the bimolecular reaction $\mathrm {S}_{2} + \mathrm {S}_{2} \overset {k_{c}}{\rightarrow } \mathrm {S}_{3}$ in the slow subsystem, the propensity is *a*_*bi*_=*k*_*c*_*x*_2_(*x*_2_−1). When *x*_2_<0, *a*_*bi*_ is positive which can potentially cause the reaction to fire and further decrease the value of *x*_2_. Thus, the SSA system becomes unstable when *x*_2_<0.

For the fast ODE system, we have

$$\begin{array}{*{20}l} \frac{dx_{1}}{dt} &= -f_{1}x_{1} + b_{1} x_{2}^{2} \\ \frac{dx_{2}}{dt} &= 2f_{1}x_{1} - 2b_{1} x_{2}^{2} \end{array} $$

The Jacobian matrix is

$$J = \left [ \begin{array}{c c} -f_{1} & 2b_{1}x_{2} \\ 2f_{1} & -4b_{1}x_{2} \end{array} \right] $$

The determinant is

$$\begin{array}{*{20}l} |\lambda I- J| &= \left | \begin{array}{c c} \lambda+f_{1} & -2b_{1}x_{2} \\ -2f_{1} & \lambda+4b_{1}x_{2} \\ \end{array} \right | \\ & = \lambda(\lambda+f_{1}+4b_{1}x_{2}) \end{array} $$

The two eigenvalues of the ODE system are *λ*_1_=0, *λ*_2_=−*f*_1_−4*b*_1_*x*_2_. For *x*_2_>0, *λ*_2_<0, so the fast system is stable. But in the HR hybrid method, *x*_2_ could be negative under certain conditions. Let $\frac {dx_{2}}{dt} = 2f_{1}x_{1} - 2b_{1} x_{2}^{2}=0$, species S_2_ has the two equilibrium points:

21 $$ x_{2}^{*} = -a \pm \sqrt{a^{2}+am}, \quad a=\frac{f_{1}}{4b_{1}}  $$

where *m* is the initial total population. One equilibrium point is positive, thus it is a stable point in the system. But for the other point, since $x_{2}^{*} = -a - \sqrt {a^{2}+am} < -2a < -\frac {f_{1}}{4b_{1}}$, *λ*_2_>0, it is an unstable point. So the HR hybrid method fails in this nonlinear system when the population of S_2_ gets smaller than $-\frac {f_{1}}{4b_{1}}$ by chance.

**Fig. 10:**
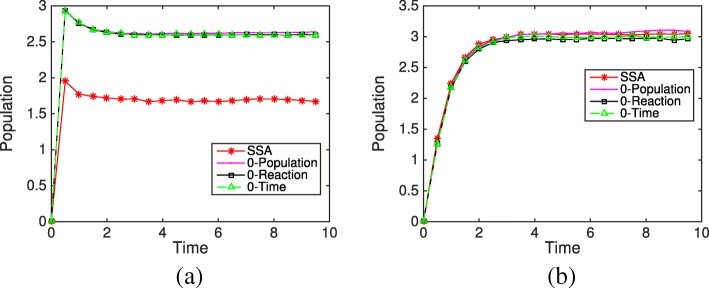
Evolution of species S_2_ and S_3_ in the nonlinear system (20) from the SSA and three remedies (Zero-Population, Zero-Reaction, and Zero-Time) based on 10,000 simulations. The parameters are *f*_1_=*b*_1_=*k*_*c*_=*k*_*s*_=1. The initial condition is *m*_1_=10 and the remaining species populations are zero. **a** Population evolution of species S _2_**b** Population evolution of species S _3_

**Fig. 11:**
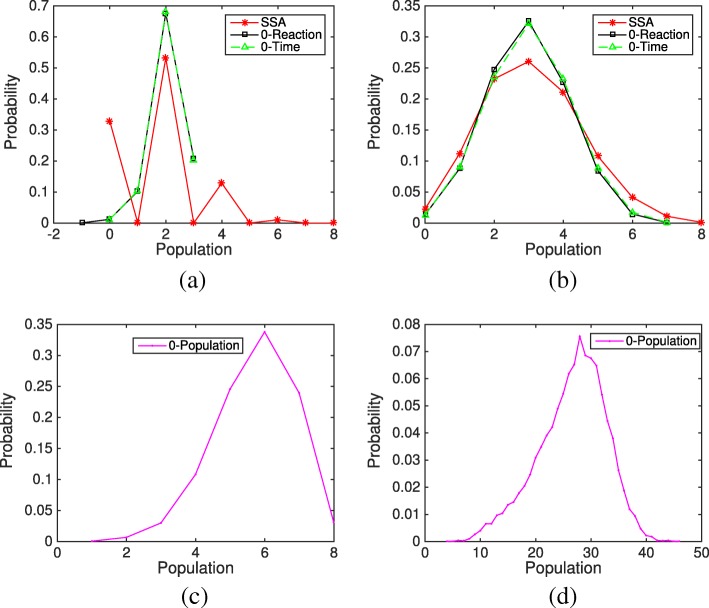
Final distributions of species S_2_ and S_3_ in the nonlinear system (20) from the SSA, three remedies (Zero-Population, Zero-Reaction, and Zero-Time) based on 10,000 simulations. The parameters are *f*_1_=*b*_1_=*k*_*c*_=*k*_*s*_=1. The initial condition is *m*_1_=10 and the remaining species populations are zero. **a** Final population distributions of species S _2_**b** Final population distributions of species S _3_**c** Final population distribution of species S _2_ under the Zero-Population remedy **d** Final population distribution of species S _3_ under the Zero-Population remedy

The above analysis is consistent with our simulation results. In our experiments, a simulation is considered a failure when the ODE solver is unable to meet integration tolerances with the smallest step length, or when one of the species’ population reaches an extremely abnormal value, for example if a species’ population becomes abnormally large (e.g. 1000) or below the negative value of the total population (−*m*). In Fig. 10, with an initial condition *m*_1_=10 and *m*_2_=0, it is found that even if *x*_2_ has a probability less than 1% to be negative (see Fig. 11a), the system still suffers a significant error and breaks down after certain simulation time when using the original HR hybrid method. Particularly, 191 simulations of the original HR hybrid method failed among the total 10,000 trials (each trial runs from time *t*=0 to *t*=10). In Fig. 10, while the evolution of S_3_ from the three rules is pretty close to that of the SSA, there is an approximate one molecule difference in the S_2_ population between the remedy rules and the SSA, which mainly comes from the method error rather than the influence of negative value of *x*_2_. The final distributions of species S_3_ from the Zero-Reaction and Zero-Time rules are close to the bell shape of the SSA results. Although the Zero-Population remedy did not fail in the simulation, the results are quite erroneous. Note that in Fig. 10, the population of S_2_ and S_3_ from the Zero-Population rule does increase slowly with time. If we run the system to a much larger time (e.g. *t*=1000), S_3_ can reach 30, as shown in the final distribution of Fig. 11d.

**Fig. 12:**
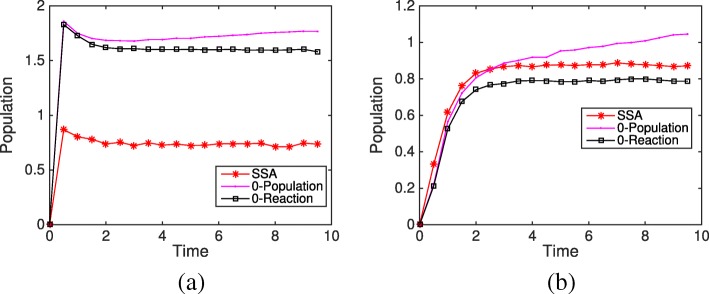
Evolution of species S_2_ and S_3_ in the nonlinear system (20) from the SSA, the HR hybrid method, and three remedies (Zero-Population, Zero-Reaction, and Zero-Time) based on 10,000 simulations. The parameters are *f*_1_=*b*_1_=*k*_*c*_=*k*_*s*_=1. The initial condition is *m*_1_=3 and the remaining species populations are zero. **a** Population evolution of species S _2_**b** Population evolution of species S _3_

**Fig. 13:**
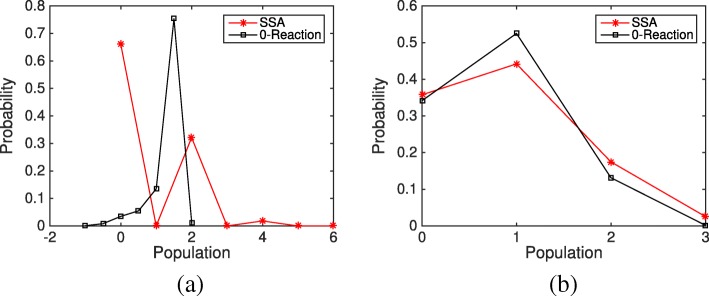
Final distributions of species S_2_ and S_3_ in the nonlinear system (20) from the SSA, the Zero-Reaction remedy based on 10,000 simulations. The parameters are *f*_1_=*b*_1_=*k*_*c*_=*k*_*s*_=1. The initial condition is *m*_1_=3 and the remaining species populations are zero. **a** Final population distributions of species S _2_**b** Final population distributions of species S _3_

When decreasing the total population to *m*=3, only the Zero-Reaction rule still works and generates stable results similar to the SSA except the approximate one molecule difference in S_2_ population, see Figs. 12 and 13. The Zero-Time rule failed because the separate G*f*′ system (which is the fast subsystem in this case) is unstable. Among the 10,000 simulations where the system was simulated from time *t*=0 to *t*=10, the original HR hybrid method failed in 2468 trials, while the Zero-Time rule failed in 1302 trials.

In general, the system stability will be affected if negative species are involved in nonlinear reactions where either the corresponding reacting terms in the ODE system or the corresponding propensities in the SSA system are still positive. Through the above comparison, the Zero-Reaction rule shows its ability to avoid the instability of nonlinear systems caused by negative values and at the same time keeps the accuracy of HR hybrid method. In application, it is easy and efficient to implement.

#### *Caulobactor crescentus* cell cycle model

*Caulobactor crescentus* is a bacteria that lives in freshwater like streams and lakes. It has an asymmetrical division that produces two morphologically different daughter cells, which makes it an important study organism for cell cycle modeling. Li et al. [31] studied the stochastic spatiotemporal model of a response-regulator network in the cell cycle. The stochastic model focused on the bistable switch of PleC that functioned as both kinase and phosphatase and successfully captured the viability of mutant cells. But it took the stochastic simulation three days for a single run.

To improve the efficiency, we applied the HR hybrid method and compared different partitioning strategies. In the stochastic model, there are 141 reactions involving 45 species, in which eight proteins and their corresponding mRNAs are diffusive. The rod cell shape was modeled as 50×1×1 cubics. In a test run, protein diffusion took 99.826*%* of the total number of reactions, putting it in the ODE system would greatly decrease the time cost. The catalytic reactions CtrA $\mathop {\rightleftharpoons }$ CtrAp took 0.148*%*. Compared to the diffusion of proteins, the diffusion of mRNAs only occupied 0.024*%*, while the remaining 140 reactions took 0.002*%*.

The computational cost of the hybrid method is proportional to the number of slow reaction firings, but is also affected by the size of the ODE subsystem. Based on the firing number of different reactions in the SSA simulation, we investigated three partitioning strategies as shown in Table 1. In Strategy I, only diffusion events of eight proteins are simulated by the ODE subsystem, the remaining events are put into the SSA subsystem. While Strategy II further partitions catalytic reactions of CtrA into the ODE subsystem, the size of the ODE subsystem does not change (reactants and products of the catalytic reaction are diffusive and already included in the ODE subsystem). But the average slow reaction firing time is one order of magnitude less than Strategy I, which decreases the time cost by approximately a factor of ten. Strategy III partitions the system by species type: mRNA reactions are all in the SSA subsystem and protein reactions are in the ODE subsystem. This strategy greatly reduces the probability of negativity problems. On the other hand, although Strategy III has the least interruption by slow reactions (every 6*e*^−5^ min), its size for the ODE subsystem increases to 50 (bin number) × 37 (types of species). This is quite a large ODE system, which imposes a high computational burden on the ODE solver. Overall, Strategy II is the most efficient partitioning strategy for the HR hybrid method in this *Caulobactor* cell cycle model, the time cost for a single cell cycle simulation is significantly reduced to one hour from three days!

**Table 1:** Comparison of different partition strategies and complexities

		Strategy I	Strategy II	Strategy III
ODE	Reactions	Protein diffusion	Protein diffusion, catalytic reaction	Diffusion, reactions involving proteins
	System Size	50 × 8 equations	50 × 8 equations	50 × 37 equations
SSA	Reactions	mRNA diffusion, all 141 reactions	mRNA diffusion, rest 140 reactions	mRNA diffusion, synthesis, degradation
	Firing Interval	1*e*^−6^	2*e*^−5^	6 *e*^−5^
Running Time of HR hybrid Method		9.5h	1h	4h

**Fig. 14:**
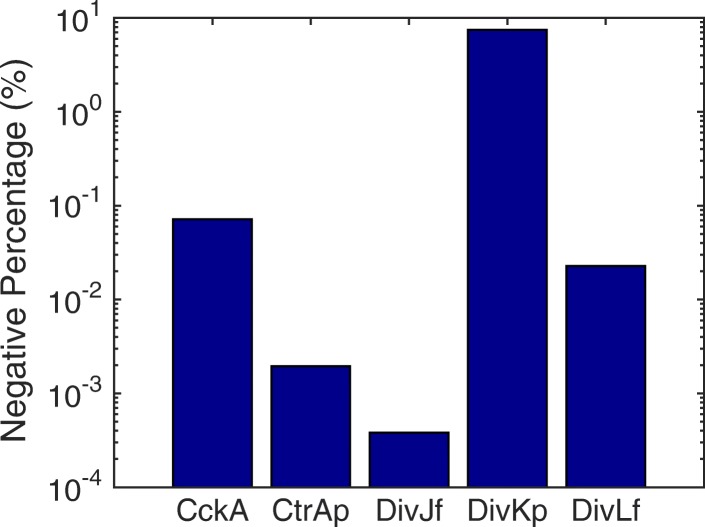
The percentage of the cell cycle time where species have negative populations

**Fig. 15:**
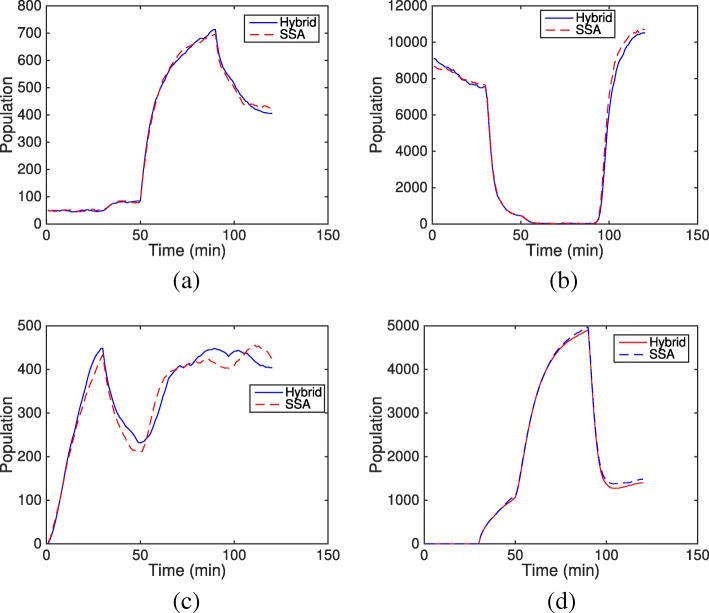
The mean population trajectories of negative species in the *Caulobactor* cell cycle model from the SSA and the HR hybrid method over 48 simulations. Note that the shown population of each species at each time point are the summation of the population over 50 bins in the domain. **a** Average population trajectories of species CckA **b** Average population trajectories of species CtrAp **c** Average population trajectories of species DivJf **d** Average population trajectories of species DivKp

However, as mentioned in the second example in the introduction, the negativity problem appears when species density is low. Figure 14 summarizes the total time of negativity state for each species during one cell cycle (∼ 120 min). It is observed that protein DivKp has a negative value for almost 10% of a cycle period, followed by proteins CckA, DivL(free), CtrAp, and DivJ(free), which are negative for less than 0.1*%* of the total time. Figure 15 shows the average population trajectories of four negative species from the SSA and the HR hybrid method using Strategy II. We can see that the hybrid method matches well with the SSA except for a slight difference in DivJ(free). It is also found that all species with negative values have a period of a low population during the cell cycle. The scarce density of DivKp (nearly zero for the initial 30 min) results in a high occurrence of negative value. Yet the negativity problem in this model has no significant impact on simulation accuracy because the diffusion of proteins happens much faster than chemical reactions (at least one order of magnitude faster). Whenever a bin has a negative population resulting from a slow reaction firing, proteins in neighboring bins (with positive populations) quickly diffuse to the negative bin in the ODE system and make it positive before any chemical reaction happens. The HR hybrid method does not even need a remedy rule for the negativity problem in this case. But in general, the Zero-Reaction rule is recommended since the added computational cost is minimal but the potential impacts of the negativity problem can be avoided.

## Conclusion

This paper presents an analysis on the negativity problem of the HR hybrid stochastic simulation algorithm. Based on the second slow reaction firing time, the error caused by negative populations is shown to be negligible compared to the approximation error of the method itself. In the linear chain system, the negativity phenomenon actually helps to increase the method’s accuracy. But for nonlinear systems, negative values may lead to system failure. Three remedies for negativity are proposed and studied in the context of SSRFT where Zero-Time and Zero-Reaction rules have acceptable accuracy. Particularly, the Zero-Reaction remedy can handle both extreme negative cases and nonlinear systems whereas the other two methods may fail. Without any remedy for negative populations, the HR hybrid method may still be successfully applied to a real biological network and significantly improves the efficiency via an optimized partition strategy. Overall, we conclude that the negativity phenomenon does not influence the biochemical network unless the negative species are involved in nonlinear reactions that generate positive reacting terms or propensities. In general, the Zero-Reaction remedy is recommended due to easy implementation and minimal additional computational cost.

## References

[1] Gillespie DT (1976). A general method for numerically simulating the stochastic time evolution of coupled chemical reactions. J Comput Phys.

[2] Gillespie DT (1977). Exact stochastic simulation of coupled chemical reactions. J Phys Chem.

[3] Gibson MA, Bruck J (2000). Efficient exact stochastic simulation of chemical systems with many species and many channels. J Chem Phys.

[4] Cao Y, Li H, Petzold L (2004). Efficient formulation of the stochastic simulation algorithm for chemically reacting systems. J Chem Phys.

[5] McCollum JM, Peterson GD, Cox CD, Simpson ML, Samatova NF (2006). The sorting direct method for stochastic simulation of biochemical systems with varying reaction execution behavior. Comput Biol Chem.

[6] Li H, Petzold L (2006). Logarithmic Direct Method for discrete stochastic simulation of chemically reacting systems.

[7] Slepoy A, Thompson AP, Plimpton SJ (2008). A constant-time kinetic Monte Carlo algorithm for simulation of large biochemical reaction networks. J Chem Phys.

[8] Anderson DF (2007). A modified next reaction method for simulating chemical systems with time dependent propensities and delays. J Chem Phys.

[9] Gillespie DT (2001). Approximate accelerated stochastic simulation of chemically reacting systems. J Chem Phys.

[10] Davis MHA (1984). Piecewise-Deterministic Markov Processes: A General Class of Non-Diffusion Stochastic Models. J R Stat Soc Ser B Methodol.

[11] Haseltine EL, Rawlings JB (2002). Approximate simulation of coupled fast and slow reactions for stochastic chemical kinetics. J Chem Phys.

[12] Rao CV, Arkin AP (2003). Stochastic chemical kinetics and the quasi-steady-state assumption: Application to the Gillespie algorithm. J Chem Phys.

[13] Cao Y, Gillespie DT, Petzold LR (2005). Avoiding negative populations in explicit Poisson tau-leaping. J Chem Phys.

[14] Franz U, Liebscher V, Zeiser S (2012). Piecewise-Deterministic Markov Processes as limits of markov jump processes. Adv Appl Probab.

[15] Jahnke T, Kreim M (2012). Error bound for piecewise deterministic processes modeling stochastic reaction systems. Multiscale Model Simul.

[16] Cao Y, Gillespie DT, Petzold LR (2005). The slow-scale stochastic simulation algorithm. J Chem Phys.

[17] Cao Y, Gillespie DT, Petzold LR (2005). Multi-scale stochastic simulation algorithm with stochastic partial equilibrium assumption for chemically reacting systems. J Chem Phys.

[18] Sanft KR, Gillespie DT, LRP (2011). Legitimacy of the stochastic Michaelis-Menten approximation. IET Syst Biol.

[19] Thomas P, Straube AV, Grima R (2011). Communication: Limitations of the stochastic quasi-steady-state approximation in open biochemical reaction networks. J Chem Phys.

[20] Salis H, Kaznessis Y (2005). Accurate hybrid stochastic simulation of a system of coupled chemical or biochemical reactions. J Chem Phys.

[21] Liu Z, Pu Y, Li F, Shaffer CA, Hoops S, Tyson JJ (2012). Hybrid modeling and simulation of stochastic effects on progression through the eukaryotic cell cycle. J Chem Phys.

[22] Lecca P, Bagagiolo F, Scarpa M (2017). Hybrid deterministic/stochastic simulation of complex biochemical systems. Mol BioSyst.

[23] Lo WC, Zheng L, Nie Q (2016). A hybrid continuous-discrete method for stochastic reaction-diffusion processes. R Soc Open Sci.

[24] Wang S, Chen M, Watson LT, Cao Y (2017). Efficient implementation of the hybrid method for stochastic simulation of biochemical systems. J Micromech Mol Phys.

[25] Chiam KH, Tan CM, Bhargava V, Rajagopal G (2006). Hybrid simulations of stochastic reaction-diffusion processes for modeling intracellular signaling pathways. Phys Rev E.

[26] Rossinelli D, Bayati B, Koumoutsakos P (2008). Accelerated stochastic and hybrid methods for spatial simulations of reaction–diffusion systems. Chem Phys Lett.

[27] Salis Howard, Sotiropoulos Vassilios, Kaznessis YiannisN (2006). BMC Bioinformatics.

[28] Hoops S, Sahle S, Gauges R, Lee C, Pahle J, Simus N (2006). COPASI–a complex pathway simulator. Bioinformatics.

[29] Wang S, Ahmadian M, Chen M, Tyson JJ, Cao Y (2016). A Hybrid Stochastic Model of the Budding Yeast Cell Cycle Control Mechanism. Proceedings of the 7th ACM International Conference on Bioinformatics, Computational Biology, and Health Informatics. BCB ’16.

[30] Chen M, Wang S, Cao Y. Accuracy analysis of hybrid stochastic simulation algorithm on linear chain reaction systems. Bull Math Biol. 2018. 10.1007/s11538-018-0461-z. 10.1007/s11538-018-0461-z 29992454

[31] Li F, Subramanian K, Chen M, Wang S, Cao Y (2016). A stochastic spatiotemporal model of a response-regulator network in the *Caulobacter crescentus* cell cycle. Phys Biol.

[32] Wang S, Cao Y (2015). The abridgement and relaxation time for a linear multi-scale model based on multiple site phosphorylation. PLoS ONE.

